# Near-isogenic soybean lines carrying Asian soybean rust resistance genes for practical pathogenicity validation

**DOI:** 10.1038/s41598-020-70188-7

**Published:** 2020-08-06

**Authors:** Takeshi Kashiwa, Yukie Muraki, Naoki Yamanaka

**Affiliations:** Biological Resources and Post-harvest Division, Japan International Research Center for Agricultural Sciences (JIRCAS), 1-1 Ohwashi, Tsukuba, Ibaraki 305-8686 Japan

**Keywords:** Biotic, Plant breeding

## Abstract

Asian soybean rust caused by the fungal pathogen *Phakopsora pachyrhizi* is the most devastating disease of soybean. The host cultivar specificity of the pathogen shows considerable differentiation depending on the area and season of its emergence. Although resistance genes for *P. pachyrhizi* (*Rpp*) have been reported in several soybean varieties, the genetic background of these varieties is highly differentiated. Furthermore, some of the varieties harbor unknown genetic factors in addition to *Rpp* that could influence resistance reactions against the pathogen. In order to gain a comprehensive understanding of *Rpp*–*P*. *pachyrhizi* interactions, homogenous plant material harboring *Rpp* genes is necessary. In this study, we bred *Rpp*-near isogenic lines (*Rpp*-NILs), which retained identical plant characters originating from a single genetic background, and accordingly showed low-variant compatible/incompatible reactions against the pathogen. These *Rpp*-NILs can be used as genetic resources for studying *P*. *pachyrhizi* epidemiology and elucidating resistance mechanisms. Compatible/incompatible relationships between the soybean rust resistance gene *Rpp* and isolates of the pathogen *P. pachyrhizi* are clearly distinguishable using the *Rpp*-NILs bred in this study.

## Introduction

Asian soybean rust (ASR) is the most devastating disease of soybean production worldwide. The disease is caused by the obligate biotrophic fungal pathogen *Phakopsora pachyrhizi* Sydow and Sydow^1,2^. Currently, the main strategy for control the disease is the use of fungicides^3^. Recently, however, pathogen resistance to fungicides has frequently been reported^1,4^. Genes conferring resistance against *P. pachyrhizi* (*Rpps*) have also been used to protect soybean from the disease, with eight *Rpp* loci being reported from soybean and introduced into soybean varieties to date^5–11^. For some of these *Rpp*s, several alleles have reported^11^; for example, *Rpp2* has been detected in PI 230970^12,13^ and Iyodaizu B^14^, and *Rpp3* has been reported from FT2 (PI 628932)^15^, PI 462312^12,16^, and Hyuuga^17,18^. Breeding for ASR-resistant soybean by introducing *Rpp*s is also emerging as a major strategy for disease control. Pyramiding of two or more *Rpp*s into a single variety has produced plants showing strong resistance against the pathogen^19,20^, and in this regard, several soybean varieties have been used as a source of *Rpp*s. These varieties are also used for inoculation tests to distinguish the host specificity of *P. pachyrhizi* isolates^21,22^. A set of ASR-resistant varieties are determined as “*Rpp* differentials” that possess a single *Rpp* gene. However, in some cases, differential varieties exhibit unclear susceptible/resistant reaction against the pathogens, which indicates that the genetic background of some of the differential varieties influences the susceptible/resistant phenotype^19,23^. Furthermore, plant characters such as growth rate and size of leaves differ among *Rpp* differentials, given that these varieties originate from different countries (Japan, China, India, Indonesia, and Brazil^21,22^).

In plant–pathogen interactions, a pathogen gene corresponds to a plant resistance gene, referred to as an “avirulence gene,” based on Flor’s gene-for-gene concept^24,25^. In the relationship between soybean and *P. pachyrhizi*, however, pathogen avirulence genes against *Rpp*s have yet to be identified. Nevertheless, sequences of the *P. pachyrhizi* secretome^26^ and genome (https://mycocosm.jgi.doe.gov/Phapa1, accessed on January 28, 2020) have recently been released, and this will accelerate the quest to identify pathogenicity-related genes from *P. pachyrhizi*. With respect to gaining a more comprehensive understanding of the soybean–*P. pachyrhizi* interaction, differences in plant characters and genetic background among the differential varieties appear to be bottlenecks currently hampering research efforts. In this regard, it is desirable to generate genetically uniform materials to compare results of inoculation tests.

In this study, we sought to prepare genetically homogeneous plant materials harboring *Rpp*s, for which we generated near-isogenic lines (NILs) of *Rpp*s bred from a single genetic background. Given that these NILs retain an almost identical genome, the phenotypes of the different NILs only reflects the effect of the introduced *Rpp* gene. In the present study, we bred NILs for *Rpp1*, *Rpp2*, *Rpp3*, *Rpp4*, *Rpp6*, and *Rpp1*-*b* from the ASR-susceptible soybean variety BRS 184, which shows low valiant inoculation results compared with those from other *Rpp* donor varieties. These clear reactions are useful for assessing the diversity and host-specificity of pathogens based on inoculation tests. By using our leaf-culture inoculation method^22^ and these *Rpp*-NILs, we are able to ascertain a pathogen’s compatibility/incompatibility against each *Rpp* in only 2 weeks. Moreover, these NILs are potentially applicable for the study of gene-for-gene relationship to identify avirulence genes from *P. pachyrhizi* that correspond to *Rpp*s.

## Results and discussion

### Development of *Rpp*-NILs

Nine *Rpp*-NILs (BC_5_F_2_ lines) carrying single *Rpp* genes of donor ASR-resistant varieties were successfully developed by backcrossing and SSR marker screening. The results of marker-screening from generation F_1_ to BC_5_F_2_ are shown in Table S1. In the BC_n_F_1_ from the backcross of BC_n-1_F_1_, 50% of the screened plants should carry the target *Rpp* in the heterozygous state, whereas in the BC_5_F_2_ generation, 25% of individuals should carry a homozygous target *Rpp*. In practice, we obtained frequencies of positive plants that were lower than these theoretical frequencies, owing crossing failure and recombination between markers of the target *Rpp*. In the most cases, however, an ideal number of positive plants were obtained (Table S1).

We observed that the *Rpp*-NILs retained uniform plant characters such as seed color and size, whereas the *Rpp* donor varieties are completely different from each other (Fig. S1). The *Rpp*-NILs shares more than 95% of the genomic region of BRS 184 by virtue of a single cross and five backcrosses. In order to compare the severity of infection with *P*. *pachyrhizi* isolates, these NILs and the *Rpp* donor varieties were subjected to an inoculation test.

### Disease reactions of *Rpp*-NILs

**Figure 1:**
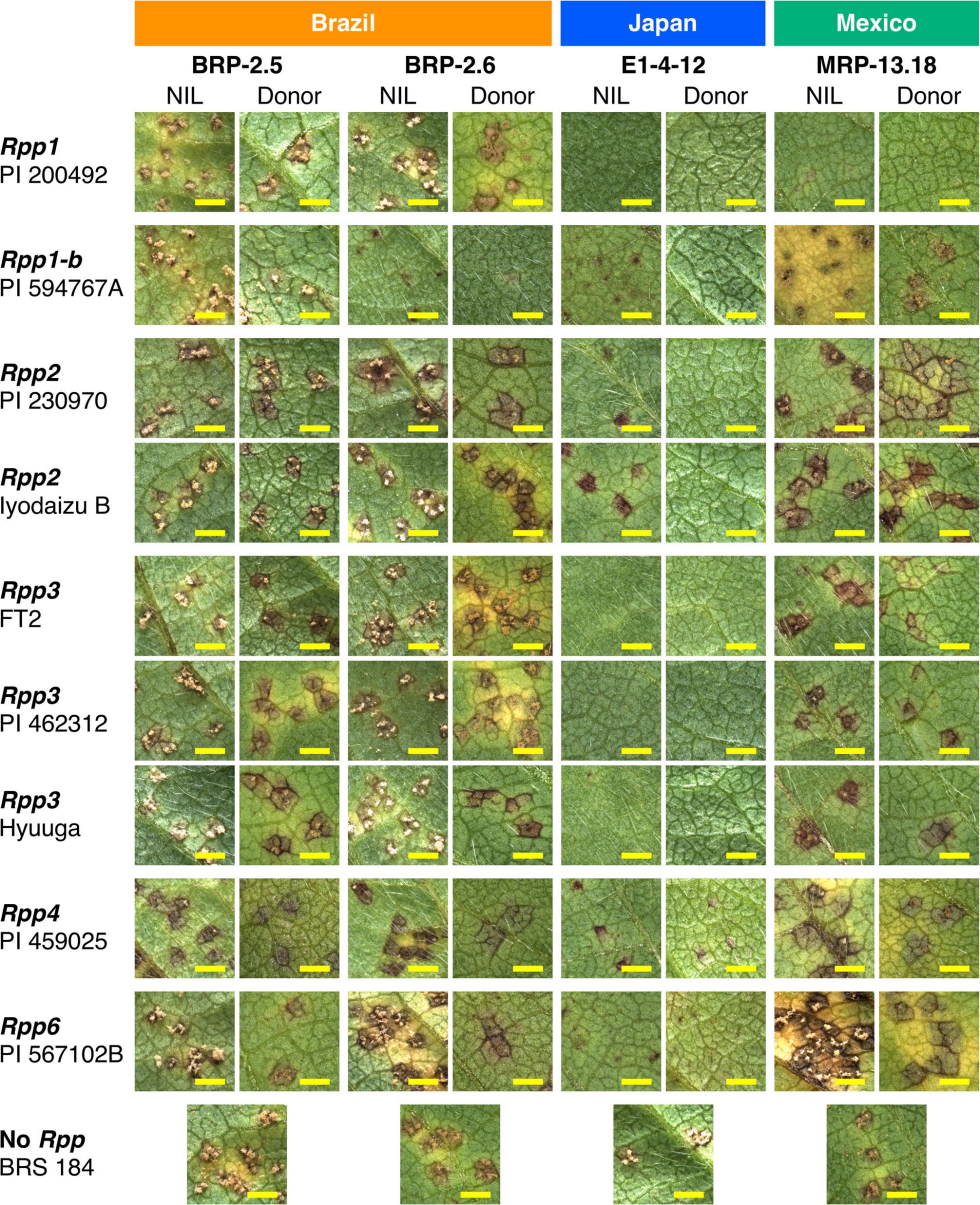
Sporulation of *Phakopsora pachyrhizi* on *Rpp*-NILs, recurrent parent, and *Rpp* donor varieties. Photographs of segments of the abaxial side (spore-inoculated side) of the leaflets of *Rpp*-NILs (NIL), recurrent parent (BRS 184) and *Rpp* donor varieties (Donor) inoculated with the four *P*. *pachyrhizi* isolates BRP-2.5, BRP-2.6, E1-4-12, and MRP-13.18. Bars = 1 mm. The photographs were taken before removing spores from the leaves.

**Figure 2:**
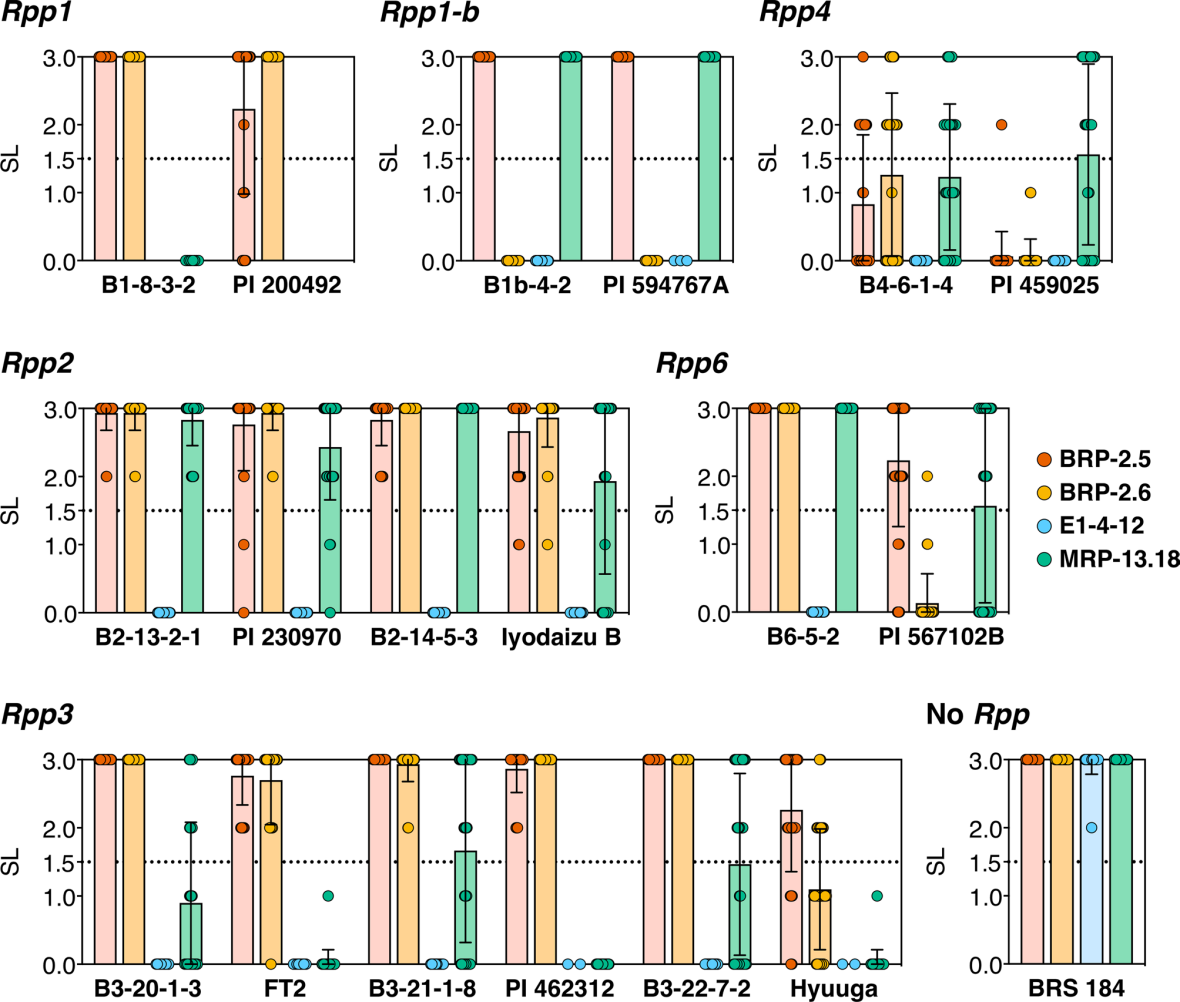
Sporulation levels on *Rpp*-NILs, recurrent parent, and *Rpp* donor varieties inoculated with *Phakopsora pachyrhizi*. Average sporulation levels (SL) of *P*. *pachyrhizi*-inoculated leaflets. The average SL values were calculated from up to 30 lesions on leaflets. An average SL value of less than 1.5 was taken to be indicative of a resistant reaction (below the dotted line). Plots on the graph show the SL of assessed lesions. Red, BRP-2.5 (Brazil); orange, BRP-2.6 (Brazil); blue, E1-4-12 (Japan); green, MRP-13.18 (Mexico). Bars indicate the standard deviation (SD).

Before determining the results of the inoculation test, we assessed germination of the spores of each *P. pachyrhizi* isolate by slide culture, and accordingly obtained the following germination percentages: 75.5% (BRP-2.5, isolate from Brazil), 86.2% (BRP-2.6, Brazil), 77.0% (E1-4-12, Japan), and 85.1% (MRP-13.18, Mexico). Among all the assessed *P. pachyrhizi* isolates, the highest level of spores developed on BRS 184 (Figs. 1, 2).

Sporulation on the *Rpp*-NILs and *Rpp* donor varieties is shown in Figs. 1 and 2. In most cases, *Rpp*-NILs showed a lower variant level of sporulation against each of four *P. pachyrhizi* isolates than the respective donor varieties (Figs. 1, 2). For example, we observed differences in sporulation level on the *Rpp1* donor variety PI 200492 and the NIL B1-8-3-2. Moreover, we observed that the donor variety infected with the Brazilian isolate BRP-2.5 showed differences in the levels of sporulation. However, in the case of NIL, all of evaluated lesion revealed that BRP-2.5 showed the highest level of sporulation, thereby indicating that compatible/incompatible relationships between pathogen and plant were more clearly identified using the NIL than the donor as host. In contrast, we observed that the Japanese *P. pachyrhizi* isolate E1-4-12 failed to form lesions on either NIL or donor (Fig. 2), which we accordingly interpret as indicating an immune response (see Table S2 for details). In the case of the Mexican isolate MRP-13.18, however, we observed lesions on the NIL, whereas no lesions could be detected on the donor, which tends to indicate the donor parental variety show a more complex resistance reaction based not only on *Rpp* but also other genes contributed to resistance against *P. pachyrhizi*.

For *Rpp2*, *Rpp3*, and *Rpp6*, we bred two, three and one NILs respectively, and all of the NILs exhibited lower variant sporulation levels against pathogens than the differential varieties (Fig. 2). However, in response to inoculation with MRP-13.18, *Rpp3*-NILs showed different levels of subsequent sporulation. The results obtained for *Rpp1*-NIL indicate that this NIL shows a weaker resistance reaction against the pathogens than the donor variety, which can be attributed to the fact that the genetic background of this NIL was *P. pachyrhizi* susceptible, and that the NIL lacks the benefits conferred by other genes contributed to resistance against ASR. Among the three *Rpp3* donors, Hyuuga is relatively resistant to Brazilian isolates and has been reported to carry another resistance gene^18^. The stronger resistance of Hyuuga than its NIL and the other two *Rpp3* donors is probably conferred by this additional resistance gene. One possibility explaining this variant reaction is the viability of the pathogen. The reactions of NILs reflect an effect of the introduced *Rpp* gene only, and pathogens compatible with the introduced *Rpp* will show clear viability. A similar response was observed for the *Rpp4*-NIL, for which sporulation varied by lesion. In contrast, we detected no differences between the NIL (B1b-4-2) and the differential variety (PI 594767A).

The uredinium is an essential structure required for pathogen sporulation, and the number of uredinia (NoU) and frequency of lesions with uredinia (%LU) are considered reliable indices for determining the ASR susceptibility/resistance of soybeans. As shown in Fig. S2, we observed no pronounced differences between the NoU on *Rpp*-NILs and the donor parents. However, the average of NoU on *Rpp*-NILs indicated a slightly weaker (susceptible) reaction against the pathogen compared with that of the donors (e.g., *Rpp6*, Fig. S2). As these observations were based on SL phenotype, a possible reason of this trend is the susceptible genetic background of the NILs. This tendency was also observed with respect to frequency of lesions with uredinia (%LU, Fig. S3).

On the basis of the presence of lesions, the presence of spores within lesions, SL, NoU, and %LU (Table S2)^22^, we categorized reaction type of the four *P. pachyrhizi* isolates on *Rpp*-NILs into five degrees. As shown in Table 1, Brazilian isolates (BRP-2.5 and BRP-2.6) were more aggressive than the other two isolates.

**Table 1:** Summary of the results of inoculation tests for *Rpp*-NILs and BRS 184.

*Rpp*	Lines	*Rpp* Donor	Generation^a^	*P. pachyrhizi* inoculation results^b^			
				BRP-2.5	BRP-2.6	E1-4-12	MRP-13.18
*Rpp1*	B1-8-3-2	PI 200492	BC_5_F_4_	S	S	I	HR
*Rpp1-b*	B1b-4-2	PI 594767A	BC_5_F_2_	S	HR	HR	S
*Rpp2*	B2-13-2-1	PI 230970	BC_5_F_4_	S	S	HR	S
*Rpp2*	B2-14-5-3	Iyodaizu B	BC_5_F_4_	S	S	HR	S
*Rpp3*	B3-20-1-3	FT2	BC_5_F_4_	S	S	HR	R
*Rpp3*	B3-21-1-8	PI 462312	BC_5_F_4_	S	S	HR	SR
*Rpp3*	B3-22-7-2	Hyuuga	BC_5_F_4_	S	S	HR	R
*Rpp4*	B4-6-1-4	PI 459025	BC_5_F_4_	R	R	HR	R
*Rpp6*	B6-5-2	PI 567102B	BC_5_F_2_	S	S	HR	S
–	BRS 184	–	–	S	S	S	S

^a^Subscript numbers indicate the number of backcrosses (BC_n_) and self-crosses (F_n_) in the breeding of *Rpp*-NILs.

^b^Category of reaction type (see Table S2 for details).

The genetic background of the NILs is that of the soybean variety BRS 184, which originates from Brazil^27,28^. Given that BRS 184 retains small plant size at the V2–3 growth stage, the *Rpp*-NILs bred in this study can be maintained in growth chambers, and 3 weeks of growth after sowing is sufficient to the inoculation test. Furthermore, the leaf culture inoculation method used in this study is rapid and can be used to reliably distinguish the efficacy of *Rpp* genes against pathogens, with lesions appearing on leaves in less than 2 weeks after inoculation. In combination with *Rpp*-NILs, this inoculation method represents a powerful tool for research on ASR epidemiology. Moreover, the recent release of genomic sequences of *P. pachyrhizi* will yield fundamental information relating to pathogen and homogeneous plant resources such as the *Rpp*-NILs, and will thus provide a strong incentive for further studies on ASR.

In most cases, the *Rpp*-NILs showed clear susceptible/resistant reactions against the pathogens (Figs. 1, 2), whereas in some *Rpp* differentials, genetic background influences the phenotype related to ASR susceptibility/resistance^19,23^. Given that, apart from the target *Rpp*s, *Rpp*-NILs theoretically share more than 95% similarity with respect to genetic background, these homogenous genetic resources are applicable for studies on gene-for-gene relationships between soybean *Rpp* genes and the avirulence genes of *P. pachyrhizi*.

## Materials and methods

### Development of *Rpp*-NILs

The ASR-resistant soybean varieties/lines: PI 200492 (alternative name Komata; carrying resistance gene, *Rpp1*), PI 594767A (Zhao Ping Hei Dou; *Rpp1-b*), PI 230970 (No. 3; *Rpp2*), Iyodaizu B (*Rpp2*), FT2 (*Rpp3*), PI 462312 (Ankur; *Rpp3*), Hyuuga (*Rpp3*), PI 459025 (Bing Nan; *Rpp4*), and PI 567102B (MARIF 2767; *Rpp6*) were used as donor parents of respective resistance genes to cross with an ASR-susceptible Brazilian variety, BRS 184 (Table S3). PI 200492, PI 594767A, PI230970, FT2, PI462312, PI459025, PI 567102B, and BRS 184 were provided by the Brazilian Agricultural Research Corporation (Embrapa) in Brazil. Iyodaizu B and Hyuuga were provided by the National Institute of Crop Science (NICS) in Japan. Six PI accessions and 2 Japanese varieties: Iyodaizu B and Hyuuga can be accessed via U.S. National Plant Germplasm System (https://npgsweb.ars-grin.gov/gringlobal/search.aspx) and NARO Genebank project (https://www.gene.affrc.go.jp/index_en.php), respectively. The F_1_ plants thus obtained were once again crossed with BRS 184 (backcross) to generate BC_1_F_1_ plants. In order to achieve 95% identity between NILs and BRS 184 and among NILs, we performed a total of five backcrosses. In the process of recurrent backcrossing, BRS 184 was used as an ovule parent at least once to exclude genetic influences from cytoplasmic difference between donor parents and BRS 184. BC_5_F_1_ plants were then selfed to obtain BC_5_F_2_ plants (BCF_2_). BC_5_F_3_ and BC_5_F_4_ plants were developed by single-seed decent (SSD) from BC_5_F_2_, Therefore, we consider that all NILs are BC_5_F_2_ lines. A total of nine NILs (Table 1) were developed and BC_5_F_2_ (for the NILs B1b-4-2 and B6-5-2) or BC_5_F_4_ plants (for the other 7 NILs) were used for inoculation with pathogens and evaluation of their reactions.

F_1_ plants were assessed for hybridisms using one of the simple sequence repeat (SSR) markers polymorphic between the parents. The backcrossed progenies (BC_1_F_1_–BC_5_F_1_) were also screened using SSR markers (Table S3) to determine whether they carry the target resistance genes of their donor parents in the heterozygous state. For each *Rpp* locus, we used at least two SSR markers polymorphic between the parents and the sandwiching target *Rpp* locus. BC_5_F_2_ plants were screened using the same SSR markers used in BC_5_F_1_ generation to obtain BC_5_F_2_ plants carrying resistant *Rpp* alleles in the homozygous state. Given that BC_5_F_2_ plants should possess *Rpp* genes in the homozygous state, all SSR markers used in this study were co-dominant between parents. DNA extraction, PCR amplification, and subsequent electrophoresis were performed following previously described procedures^22^.

### *Phakopsora pachyrhizi* inoculation test on *Rpp*-NILs

To evaluate the ASR compatibility/incompatibility of *Rpp*-NILs, *P. pachyrhizi* isolates from three countries, Japan (E1-4-12), Brazil (BRP-2.5 and BRP-2.6), and Mexico (MRP-13.18) were subjected to an inoculation test. Of the aforementioned isolates, E1-4-12, BRP-2.5, and BRP-2.6 were isolated in our previous studies^14,23^, whereas MRP-13.18 was isolated from MRP-13^29^ by a single-lesion isolation method^22^ in the present study. The Brazilian and Mexican *P. pachyrhizi* isolates are Import-prohibited articles in Japan where the work was conducted (Import Permit Numbers: 20Y157 and 27Y935 from the Yokohama Plant Quarantine Office of Japan for Brazilian and Mexican *P. pachyrhizi* populations, respectively). Spores of *P. pachyrhizi* collected from soybean leaves were dried and maintained at − 80 °C for long-term storage. Frozen spores were heated to 39 °C in a water bath for 1 min immediately prior to inoculation. Inoculation of *P. pachyrhizi* isolates was performed following an online manual^22^. Briefly, leaflets were detached from the lowest trifoliate leaf of V2–3 stage soybean plants, and gently hand-rubbed with autoclaved distilled water to remove trichomes on the leaf surface. Spores of *P. pachyrhizi* were soaked in 0.04% Tween 20 and adjusted to a concentration of 5.0 × 10^4^ spores/mL. For each inoculation, spore suspensions were spread onto three soybean leaves using a paintbrush. Each of three leaves were sampled from different plants. Inoculated leaves were maintained at 21 °C in Petri dishes to maintain moist conditions. Immediately after inoculation, the Petri dishes were covered in aluminum foil to maintain darkness for 24 h. Two weeks after inoculation, we evaluated the average sporulation level (SL), average number of uredinia per lesion (NoU), and frequency of lesions with uredinia (%LU) in all assessed lesions for each of up to 30 lesions per inoculation. We initially evaluated SL, after which NoU was counted following the removal of spores from the inoculated leaves using a paintbrush. Statistical analysis of each value was performed using GraphPad Prism 8 (GraphPad Software, San Diego, CA, USA).

## Supplementary information

Supplementary Information.

## Data Availability

Near isogenic lines (NILs) used in this study are available at Japan International Research Center for Agricultural Sciences under Material Transfer Agreement.
