# Supplementary material for: Near-isogenic soybean lines carrying Asian soybean rust resistance genes for practical pathogenicity validation
Source: Sci Rep. 2020 Aug 6;10:13270. doi: 10.1038/s41598-020-70188-7 (PMC7411041; doi:10.1038/s41598-020-70188-7)
Supplement: Supplementary file 1 — Supplementary Information. [file 41598_2020_70188_MOESM1_ESM.docx]

**Title:** Near-isogenic soybean lines carrying Asian soybean rust resistance genes for practical pathogenicity validation

**All author names:**

Takeshi Kashiwa, Yukie Muraki, and Naoki Yamanaka*

*author for correspondence: Naoki Yamanaka

Biological Resources and Post-harvest Division, Japan International Research Center for Agricultural Sciences (JIRCAS), 1-1 Ohwashi, Tsukuba, Ibaraki 305-8686, Japan

email: naokiy@affrc.go.jp

**
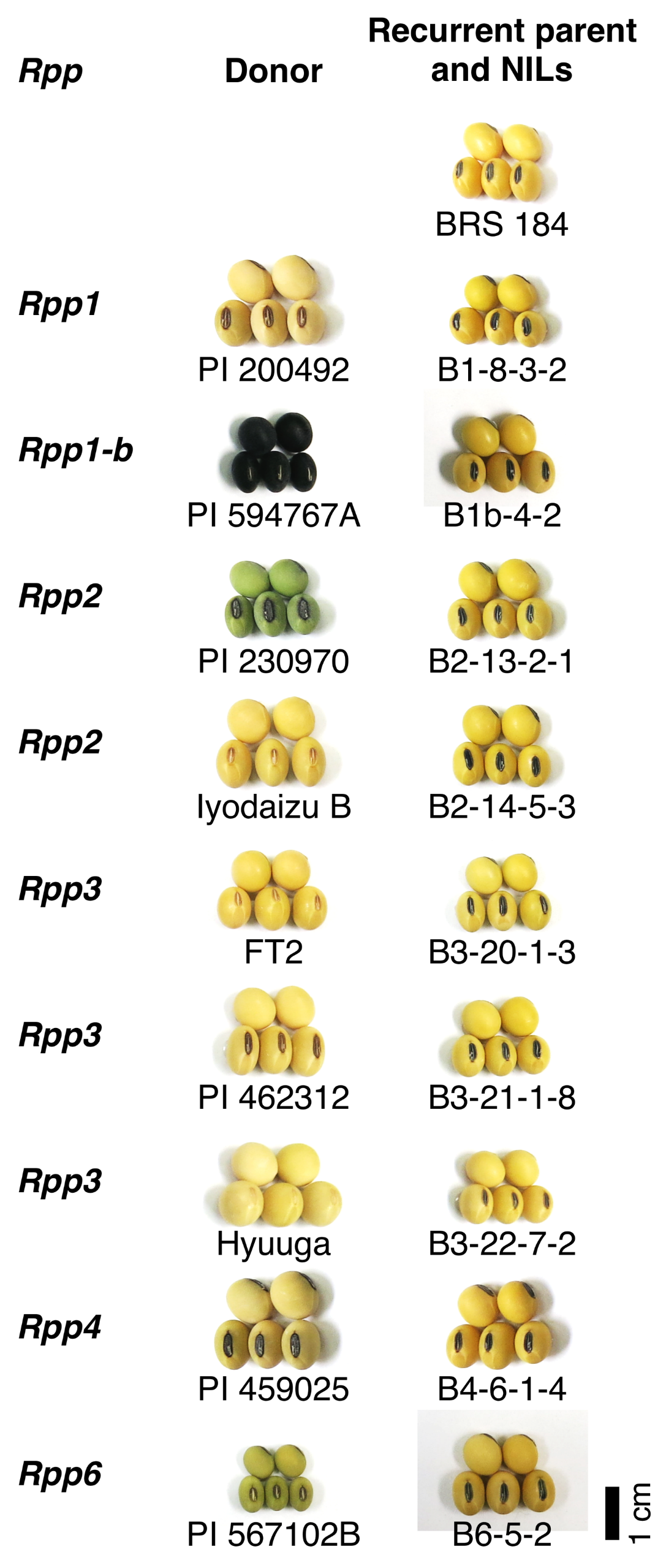
**

**Fig. S1 Appearance of the seeds of *Rpp* donors and *Rpp*-NILs**

Photographs of the seeds of *Rpp* donor varieties, recurrent parent of NILs (BRS 184), and *Rpp*-NILs. Bar indicates 1 cm
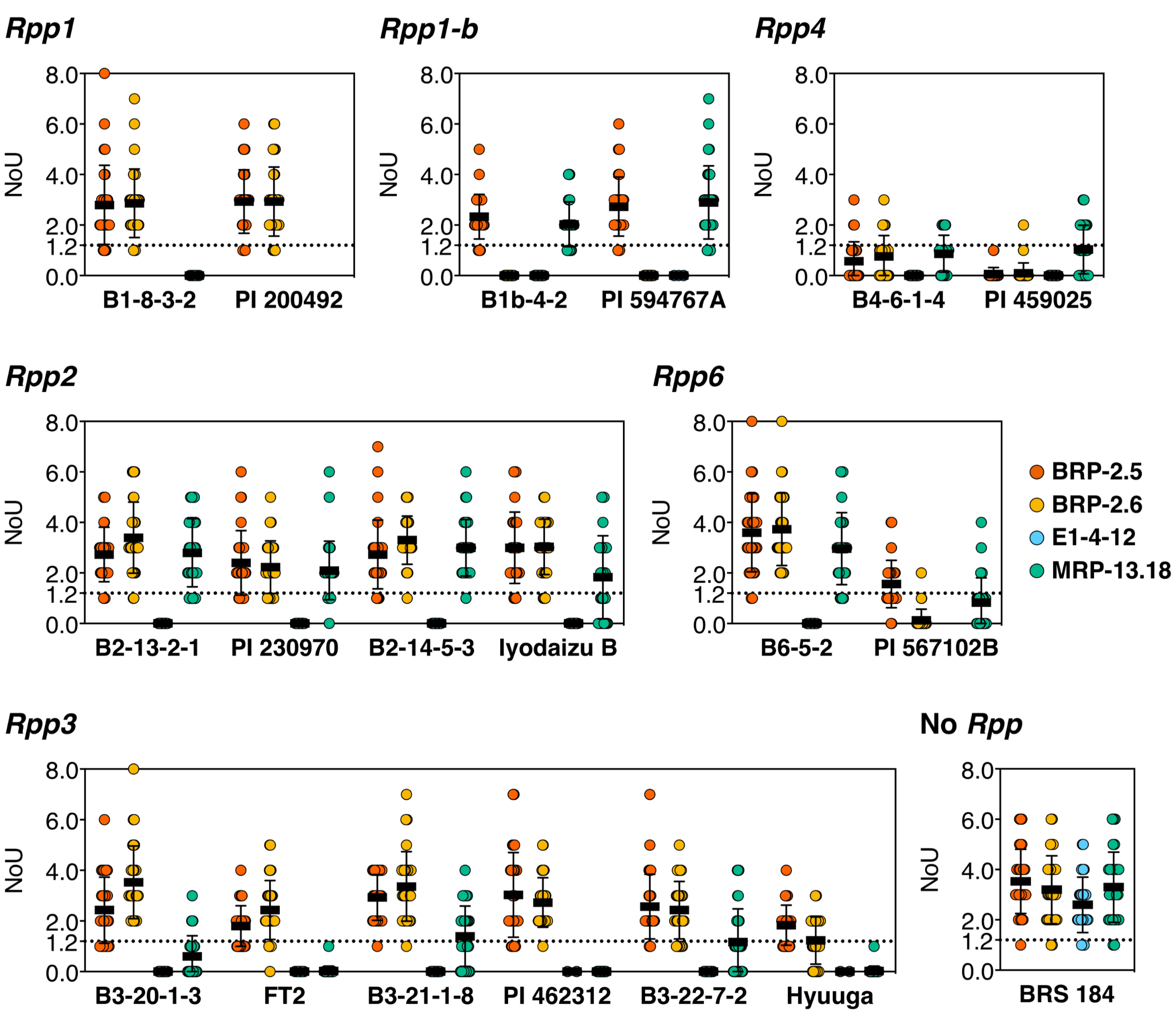


**Fig. S2 Number of uredinia per lesion on *Rpp*-NILs, recurrent parent, and *Rpp* donor varieties inoculated with *Phakopsora pachyrhizi***

Average number of uredinia per lesion (NoU) on *P*. *pachyrhizi*-inoculated leaflets. The average NoU (bold horizontal line) was calculated from up to 30 lesions on leaflets. An average NoU value of less than 1.2 was taken to be indicative of a resistant reaction (below the dotted line). Plots on the graph show the NoU of assessed lesions. Red, BRP-2.5 (Brazil); orange, BRP-2.6 (Brazil); blue, E1-4-12 (Japan); green, MRP-13.18 (Mexico). Bars indicate the standard deviation (SD)


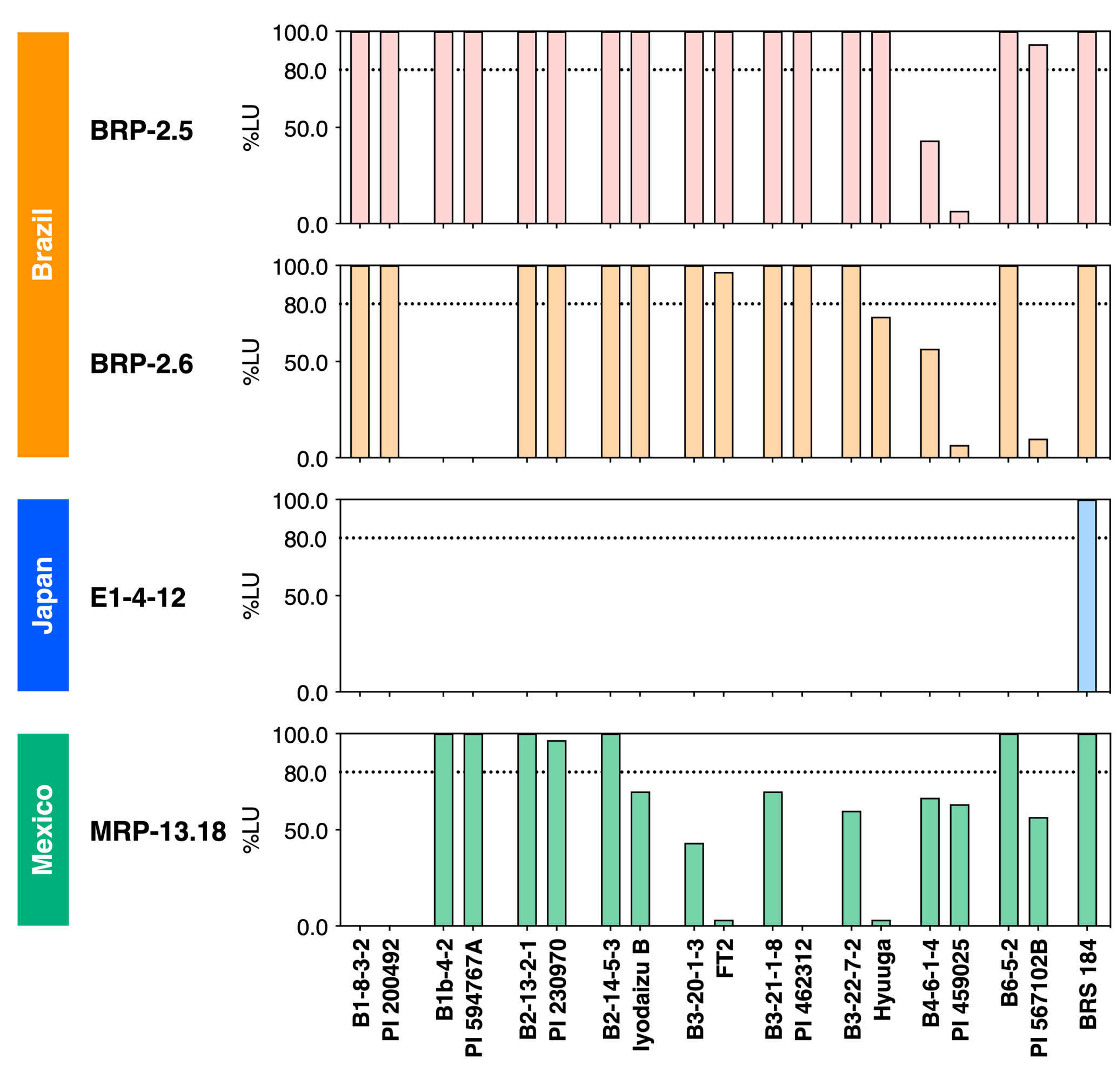


**Fig. S3 Frequency of lesions with uredinia on *Rpp*-NILs, recurrent parent, and *Rpp* donor varieties inoculated with *Phakopsora pachyrhizi***

The frequency of lesions with uredinia (%LU) was calculated from up to 30 lesions on leaflets. A %LU of less than 80.0 was taken to be indicative of a resistant reaction (below the dotted line). Pink, BRP-2.5 (Brazil); orange, BRP-2.6 (Brazil); blue, E1-4-12 (Japan); green, MRP-13.18 (Mexico). Each of *Rpp*-NILs (left) and corresponding *Rpp* donors (right) are displayed side by side

**Table S1 Frequency of positive plants carrying the target *Rpp* in each backcross generation**

| **NIL** | **Donor** | ***Rpp*** | **Positive plants^1^/Screened plants** | | | | | | |
| --- | --- | --- | --- | --- | --- | --- | --- | --- | --- |
|  |  |  | **F_1_** | **BC_1_F_1_** | **BC_2_F_1_** | **BC_3_F_1_** | **BC_4_F_1_** | **BC_5_F_1_** | **BC_5_F_2_** |
| B1-8-3-2 | PI 200492 | *Rpp1* | 3 / 3 | 2 / 4 | 2 / 6 | NA | NA | 4 / 5 | 3 / 12 |
| B1b-4-2 | PI 594767A | *Rpp1-b* | 5 / 5 | 3 / 4 | 1 / 11 | 5 / 9 | 2 / 8 | 4 / 10 | 9 / 33 ^2^ |
| B2-13-2-1 | PI 230970 | *Rpp2* | 3 / 3 | 2 / 4 | 2 / 4 | 4 / 10 | NA | 3 / 5 | 4 / 11 |
| B2-14-5-3 | Iyodaizu B | *Rpp2* | 1 / 1 | 4 / 4 | 4 / 11 | 5 / 12 | NA | 2 / 5 | 4 / 12 |
| B3-20-1-3 | FT2 | *Rpp3* | 3 / 3 | 4 / 12 | 8 / 13 | NA | NA | 2 / 5 | 4 / 11 |
| B3-21-1-8 | PI 462312 | *Rpp3* | 4 / 4 | 2 / 4 | 5 / 13 | 7 / 10 | NA | 3 / 5 | 3 / 23 |
| B3-22-7-2 | Hyuuga | *Rpp3* | 2 / 2 | 1 / 1 | 6 / 10 | 4 / 20 | NA | 4 / 11 | 2 / 12 |
| B4-6-1-4 | PI 459025 | *Rpp4* | 4 / 4 | 3 / 4 | 7 / 9 | 1 / 7 | NA | 3 / 5 | 4 / 11 |
| B6-5-2 | PI 567102B | *Rpp6* | 6 / 6 | 2 / 5 | 1 / 7 | 2 / 9 | 4 / 11 | 1 / 3 | 6 / 32  ^2^ |
| (Theoretical % of positive plants) | | | 100% | 50% | 50% | 50% | 50% | 50% | 25% |

^1^Positive plants carrying the target *Rpp* genes in the heterozygous state in the F_1_ and BC_n_F_1_ generations, and in the homozygous state in the BC_5_F_2_ generation

^2^Six positive plants were used for the experiment in this study

NA: BC_n_F_1_ plants were obtained by backcross with BC_n-1_F_2_ or BC_n-1_F_3_ plants

**Table S2 Criteria used to determine reaction type**

| **Category** | **Abbreviation** | **Lesion^1^** | **Uredinia^2^** | **SL^3^** | **NoU^4^** | **%LU^5^** |
| --- | --- | --- | --- | --- | --- | --- |
| Immune | I | – | – | – | – | – |
| Highly resistant | HR | + | – | – | – | – |
| Resistant | R | + | + | R | R | R |
| Slightly resistant | SR | + | + | S/R^6^ | S/R^6^ | S/R^6^ |
| Susceptible | S | + | + | S | S | S |

Modified from an online manual [22]. Cutoff levels for the SL, NoU, and %LU values used to determine resistance (R) are adjusted for the inoculation assay using leaf culture

^1^Presence (+) or absence (–) of lesions on leaves

^2^Presence (+) or absence (–) of uredinia within lesions

^3^Average sporulation level (SL) of lesions. SL < 1.5 indicates R

^4^Average number of uredinia per lesions (NoU). NoU < 1.2 indicates R

^5^Frequency of lesions with uredinia (%LU). %LU < 80.0 indicates R

^6^One or two R in SL, NoU, and %LU indicates slightly resistant (SR)

**Table S3 *Rpp* donor varieties and SSR markers used for selecting backcrossed plants**

| ***Rpp*** | ***Rpp* Donor** | **Accession^1^** | **Origin** | **SSR markers^2^** | **References^3^** |
| --- | --- | --- | --- | --- | --- |
| *Rpp1* | Komata | PI 200492 | Japan | Sct_187, Sat_064 | [5] |
| *Rpp1-b* | Zhao Ping Hei Dou | PI 594767A | China | Sat_064, SSR18_1900, Sat_372, Sat_117 | [8] |
| *Rpp2* | No. 3 | PI 230970 | Japan | Satt620, SSR16_0912, Satt380 | [13] |
|  | Iyodaizu B | NA | Japan | Satt620, SSR16_0912, Satt621, Satt380 | [14] |
| *Rpp3* | FT2 | (PI 628932)* | Brazil | Satt460, Sat_238, SSR06_1521, Sat_142, Sat_263 | [15] |
|  | Ankur | PI 462312 | India | Sat_238, SSR06_1530, Satt460, Sat_263 | [16] |
|  | Hyuuga | (PI 506764)* | Japan | Satt460, SSR06_1521, Sat_263 | [17] |
| *Rpp4* | Bing Nan | PI 459025 | China | Satt288, SSR18_1576, AF162283 | [6] |
| *Rpp6* | MARIF 2767 | PI 567102B | Indonesia | Satt324, SSR18_0408, Satt394 | [7] |

^1^Accessions marked by an asterisk were determined according to the origin of the variety based on the USDA website (https://npgsweb.ars-grin.gov/gringlobal/search.aspx, accessed 27 January 2020); NA, not assigned

^2^Two or three SSR markers of them were used to determine the genotypes of backcrossed progenies

^3^References of *Rpp* genes
